# Effects of matcha green tea on the pharmacokinetics of nadolol in rats

**DOI:** 10.1371/journal.pone.0342857

**Published:** 2026-02-13

**Authors:** Eslam T. Mashaqbeh, Tamam El-Elimat, Osama Y. Alshogran, Iyad Hamzeh, Zahraa M. Obeidat, Ahmed H. Al Sharie, Feras El Hajji

**Affiliations:** 1 Department of Medicinal Chemistry and Pharmacognosy, Faculty of Pharmacy, Jordan University of Science and Technology, Irbid, Jordan; 2 Department of Clinical Pharmacy, Faculty of Pharmacy, Jordan University of Science and Technology, Irbid, Jordan; 3 Department of Internal Medicine, Southeast Health, Dothan, Alabama, United States of America; 4 Department of Clinical Pharmacy and Therapeutics, Faculty of Pharmacy, Applied Science Private University, Amman, Jordan; Central University of Rajasthan, INDIA

## Abstract

The concurrent use of herbal dietary supplements with prescription medications raises safety concerns due to the potential for clinically significant interactions. Matcha, a shade-grown green tea consumed as an ultra-fine powder, is rich in catechins that may inhibit the transport of P-glycoprotein (P-gp) substrates such as nadolol. This study investigated the effects of administering single and multiple doses of matcha on the pharmacokinetics of nadolol in an *in vivo* animal model. Male Sprague-Dawley rats (*n* = 32) were randomly assigned to four groups. Group 1 (negative control) was administered normal saline followed by a single oral dose of nadolol (10 mg/kg). Group 2 (matcha single-dose) was administered a single dose of matcha (250 mg/kg) whisked in normal saline, followed by nadolol (10 mg/kg) after 30 min. Group 3 (positive control) received itraconazole (50 mg/kg), followed by nadolol (10 mg/kg) after 30 min. Group 4 (matcha multiple-dose) received matcha (250 mg/kg daily for 21 days) before administering nadolol (10 mg/kg) on day 21. Blood samples were collected at 0, 0.33, 0.66, 1, 1.5, 2, 3, 4, 5, 8 and 24 h. Nadolol concentrations in plasma were measured by a validated high-performance liquid chromatography with fluorescence detection (HPLC-FL) method. Pharmacokinetic parameters were estimated using the PK solver add-in for Microsoft Excel. To ensure quality control, caffeine, a key marker compound of matcha green tea, was quantified using HPLC with ultraviolet detection (HPLC-UV). A single oral dosage of matcha (250 mg/kg) had no statistically significant effects on the pharmacokinetics of nadolol compared to the control group (*p* > .05). Although the multiple-dose matcha group showed an increase in *C*_max_ (~45%), AUC_0-t_ (~18%), and AUC_0-∞_ (~22%) for nadolol compared to the control group, these differences were not statistically significant (*p* > .05). In contrast, the *t*½ (h) of nadolol increased significantly from 4.0 ± 1.6 in the control group to 7.7 ± 4.2 (*p* = .039) in the matcha multiple-dose group. Itraconazole co-administration significantly increased systemic exposure (AUC) of nadolol (*p* = .009), confirming the validity of the animal model. Caffeine, a key marker compound in matcha tea, was quantified at 4.18 ± 0.44% w/w of dry matcha tea powder, equivalent to 41.8 ± 4.4 mg/g. This is the first study to explore the potential pharmacokinetic interaction between matcha tea and nadolol. Single and multiple oral doses of matcha green tea had negligible effects on most pharmacokinetic parameters of nadolol, except for an increased half-life in the multiple-dose group. Further research is needed to establish the clinical relevance of this interaction before definitive recommendations on the safety of matcha tea and nadolol coadministration can be made.

## Introduction

There is a growing interest across the globe in the use of botanical products for managing various health conditions [1]. The retail sales of herbal dietary supplements in the United States totaled about $12.121 billion in 2022, exceeding $10 billion for the third consecutive year [1]. It has been estimated that herbal products are used by approximately 20% of the population and most of those individuals use such products on a routine basis [2,3]. This is translated into a growing number of the population using botanical products, with 20–30% indicating combined use of botanicals with conventional drugs, especially among patients with chronic diseases [4]. Around 70% of regular users of botanical supplements also take prescription medications and 32–61% of hospital patients have reported regular use of botanical supplements. However, less than 40% of patients reveal the use of herbal dietary supplements to their physicians or other health care professionals [5]. Such increasing numbers raise concerns about the risk of potential herb-drug interactions [6]. Moreover, botanical products are not subjected to the same rigorous regulations of safety and efficacy required for prescription drug approval. As a result, there is often incomplete knowledge regarding the interaction between botanical products and conventional drugs [4].

Matcha tea is a traditional Japanese green tea that has gained popularity in recent years due to its reported health benefits [7,8]. Matcha tea is made by grinding the leaves of shade-grown tea plants into a fine powder, which is then whisked with hot water to make a frothy, flavorful tea [7,8]. Matcha tea is rich in bioactive compounds, including catechins, flavonoids, phenolic acids, caffeine, theanine, and vitamins (Table 1) [9–13]. Matcha tea has been reported to have several health benefits, including antioxidant and anti-inflammatory properties, improved cognitive function, reduced stress and anxiety, weight loss, and improved heart health [9]. Although matcha tea is generally considered safe, it can have some potential adverse effects, especially when consumed in large amounts. For example, matcha tea may interact with certain medications, such as blood thinners [14].

**Table 1:** Quantitative composition of major biologically active constituents in matcha green tea reported in the literature.

Compound	Content/dry weight	Reference(s)
Gallic acid	39.4–423.0 *µ*g/g	Koláčková et. al. 2020 [15]
Chlorogenic acid	2640–4800 *µ*g/g	
Sinapic acid	89.2–1400.0 *µ*g/g	
Ellagic acid	38.1–371.0 *µ*g/g	
Rutin	361–2870 *µ*g/g	Koláčková et. al. 2020 [15]
Kaempferol	1.7–32.0 *µ*g/g	
Quercetin	8.4–84.9 *µ*g/g	
Catechin (C)	0.8 − 28.94 mg/g	Sayuti et al. 2021 [11], Zhou et al. 2021 [12]
Epicatechin (EC)	0.95 − 4.4 mg/g	
Epigallocatechin gallate (EGCG)	70.2 − 95.48 mg/g	
Epicatechin gallate (ECG)	8.3 − 74.48 mg/g	
Caffeine	14.4 − 65.8 mg/g	Koláčková et. al. 2020 [15], Zhou et al. 2021 [12], El-Elimat et al. 2022 [10]

Human clinical studies indicate that matcha consumption can exert measurable effects on metabolism, cognitive function, stress, mood, and sleep-related outcomes. In healthy females, short-term matcha intake (3 g/day for three weeks) increased fat oxidation and reduced carbohydrate oxidation during moderate-intensity exercise without affecting heart rate or total energy expenditure [16], and matcha beverages similarly enhanced fat oxidation and lowered respiratory exchange ratio during brisk walking without altering perceived exertion [17]. Cognitive and stress-related benefits have also been reported, including maintenance of attentional function following mild acute psychological stress after two weeks of matcha intake (2 g/day) in young adults [18], and reduced salivary *α*-amylase activity after 15 days of matcha consumption (4.5 g/day) incorporated into cookies, with effects linked to a low caffeine/EGCG-to-theanine/arginine (CE/TA) ratio [19]. In overweight and obese adults, 12-week matcha intake (2 g/day) combined with a low-calorie diet was associated with reductions in adiposity measures and favorable modulation of inflammatory and oxidative stress markers, although between-group differences were modest [10]. Additional evidence suggests broader well-being effects, as an 8-week guided tea meditation intervention incorporating matcha reduced perceived stress and improved mood in generally healthy adults [20]. With respect to sleep and longer-term cognitive outcomes, matcha intake (2.7 g/day for four weeks) did not significantly alter objective sleep parameters measured by EEG but improved subjective sleep satisfaction and emotional well-being [21], while a 12-month randomized controlled trial in older adults with cognitive decline demonstrated improved social acuity and a trend toward better sleep quality following daily matcha consumption (2 g/day), despite no significant changes in global cognitive scores [22].

Matcha has been proposed to interact with anticoagulant therapy primarily on mechanistic grounds. The most established interaction concerns warfarin, as green tea leaves contain vitamin K, which can antagonize warfarin’s inhibition of vitamin K–dependent clotting factors and reduce the international normalized ratio (INR), particularly when intake changes [23]. In contrast, potential interactions between matcha and direct oral anticoagulants (DOACs) remain theoretical and are based on experimental evidence indicating modulation of P-glycoprotein transport and, to a lesser extent, CYP3A4 activity by green tea catechins [24]. Current clinical guidance classifies green tea–DOAC interactions as low-level and unconfirmed, noting that clinically relevant effects are well established primarily for strong transporter or enzyme modulators rather than for dietary tea consumption [24].

There is a growing worldwide interest in matcha tea as a healthy nutrient with promising applications in the field of functional food or nutraceuticals [25]. Matcha tea is rich in catechins [9] and the coadministration of green tea catechins was reported to cause significant alterations in the pharmacokinetics of nadolol [26]. Nadolol is a non-selective beta-blocker that has been commonly used for several decades in the treatment of hypertension, angina, and other cardiovascular conditions [27]. Nadolol is a hydrophilic drug that is excreted primarily as a parent drug via the kidneys and does not undergo metabolism by the cytochrome P450 enzymes (CYPs) in the liver [28]. It has been demonstrated *in vitro* that nadolol is a substrate for a number of drug transporters. These include P-glycoproteins (P-gps), organic anion-transporting polypeptides (OATPs), and organic cation transporters (OCTs) [29,30]. Nadolol with its low permeability and high solubility is classified as class 3 in the Biopharmaceutics Classification System (BCS) [31]. This study aimed to examine, *in vivo*, the pharmacokinetic interaction between matcha green tea and nadolol and develop and validate a method for quantification of nadolol in rats’ plasma.

## Materials and methods

### Chemicals and instrumentations

Clearspring^©^ Japanese organic matcha green tea powder was purchased from the United Kingdom and delivered to Jordan via a local vendor. A voucher specimen (PHS-132) was deposited at the Herbarium of the Faculty of Pharmacy, Jordan University of Science and Technology, Irbid, Jordan. Matcha powder was stored at room temperature (24 °C), protected from direct sunlight, until needed for analysis. Nadolol (≥99% purity, Genochem, Spain), metoprolol (≥99% purity, Sigma Aldrich, USA), itraconazole (≥98% purity, Biosynth, Slovakia), acetonitrile (≥99.9, Fisher Scientific, USA), caffeine (≥99%, TCI, Japan), perchloric acid (70%, Fisher Scientific, USA), diethyl ether (≥99%, Sigma Aldrich, Germany), formic acid (≥98%, AZ Chem, China), dichloromethane (≥99.8%, TEDIA, USA), ammonium acetate (≥98%, Xilong Chemical Industry, China), sodium chloride (≥99.5%, Kuwait and Saudi Pharmaceutical Industries, Kuwait), and sodium hydroxide (≥98% BBC Chemical, Jordan) were used as received from the supplier.

A Shimadzu Prominence I LC-2030C 3D Plus RF-20A fluorescence detector (Shimadzu, Kyoto, Japan) was used for HPLC analysis. A vortex mixer (Labnet International, USA) was utilized for sample mixing. The Hermle Z 366K centrifuge (Hermle LaborTechnik, Germany) was employed for centrifugation. Additionally, a GYROVAP centrifuge evaporator with a cold trap (Howe, United Kingdom) was used for sample concentration.

### Animal experiment

The study included 32 male Sprague-Dawley rats, each weighing approximately 183 ± 7 g, which were obtained and housed in the animal facility of the Applied Science Private University (Amman, Jordan). The rats were housed in small plastic cages (four per cage) under controlled hygienic conditions in an air-conditioned room at a temperature of 24 °C. The rats had *ad libitum* access to filtered tap water and standard laboratory chow, on a 12:12 h light-dark cycle. Each rat was tail tagged for identification and acclimated under these conditions for at least one week before experimentation. The study protocol was approved by the Animal Care and Use Committee (ACUC) of Jordan University of Science and Technology (approval number 16/4/12/325).

**Fig 1:**
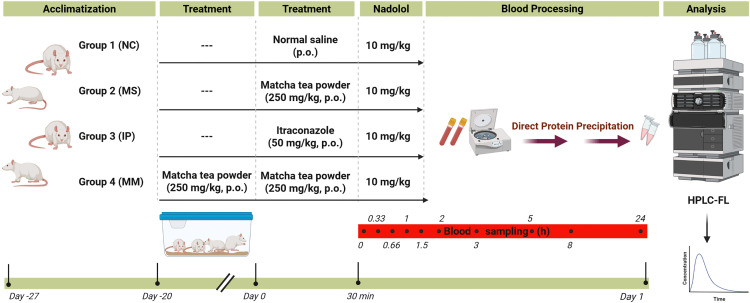
Schematic diagram of the study procedure.

Following acclimatization, the rats (*n* = 32) were randomly assigned to four groups of eight rats each (*n* = 8). The nadolol control group (NC), matcha single-dose (MS) group, itraconazole positive control group (IP), and matcha multiple-dose (MM) (Fig 1). The rats in the first group (control group) received normal saline followed 30 min later by a single oral dose of nadolol (10 mg/kg) dissolved in deionized water [30,32]. In group two (MS), a single oral dose of matcha (250 mg/kg) dissolved in normal saline was administered, which was followed 30 min later by a single oral dose of nadolol (10 mg/kg) dissolved in deionized water. Group three (IP) rats received a single oral dose of itraconazole (50 mg/kg) dissolved in normal saline [30,33], followed 30 min later by a single oral dose of nadolol (10 mg/kg) dissolved in deionized water. The rats in group 4 (MM) received matcha (250 mg/kg orally per day) for 21 days. On day 21, a single oral dose of nadolol (10 mg/kg) dissolved in deionized water was given 30 min after the last matcha dose. Doses were adjusted according to each rat’s body weight on the day of administration. The rats were fasted overnight and for three hours post-dosing, with *ad libitum* access to tap water. Blood samples were collected from the retro-orbital vein at baseline (0 h) and at 0.33, 0.66, 1, 1.5, 2, 3, 5, 8, and 24 h post-nadolol administration. Blood samples (about 0.5 mL) were collected in EDTA-coated tubes and centrifuged at 5000 rpm for 10 min to separate plasma. Plasma samples were stored at –80 °C until analyzed using high-performance chromatography with fluorometric detection.

Blood samples were collected via the retro-orbital venous plexus by trained personnel to minimize distress. At the end of the experiment, the animals were deeply anesthetized with an intraperitoneal injection of ketamine (100 mg/kg) combined with xylazine (10 mg/kg). Anesthetic depth was assessed by the absence of pedal and palpebral reflexes. Once a surgical plane of anesthesia was confirmed, animals were euthanized by cervical dislocation performed by trained personnel. Death was confirmed by cessation of respiration and lack of reflex responses. All efforts were made to minimize animal suffering and to use the minimum number of animals necessary to achieve the scientific objectives of the study.

### Determination of nadolol concentration

Stock solutions of nadolol (50 *µ*g/mL) and the internal standard (IS) metoprolol (50 *µ*g/mL) were prepared by dissolving 5 mg of accurately weighed nadolol and metoprolol standards in 100 mL of methanol/water (50/50, v/v). The standard stock solutions were mildly vortexed to ensure complete solubility. Nadolol stock solution (50 *µ*g/mL) was serially diluted using blank rat plasma to prepare seven calibration concentrations at 2.5, 5, 10, 50, 100, 500, and 1000 ng/mL. Three quality control samples were prepared in plasma at 5 ng/mL [quality control low (QCL)], 100 ng/mL [quality control medium (QCM)], 1000 ng/mL [quality control high (QCH)]. Stock solutions, calibration standards, and QC samples were stored at –80 °C. Nadolol was extracted from plasma samples using direct protein precipitation. About 10 *µ*L of metoprolol (IS) was mixed with a 100 *μ*L plasma aliquot in an Eppendorf tube, followed by the addition of 100 *μ*L of perchloric acid (10%). The mixture was vortexed for ~1 min and centrifuged at 4500 rpm for 5 min. The supernatant was then transferred to an HPLC vial, and a 60 *μ*L aliquot was injected into the HPLC system for analysis.

An HPLC-FL method was developed and validated for the quantification of nadolol in plasma samples. The mobile phase used was a gradient system that consisted of 50 mM ammonium acetate with pH adjusted by acetic acid to a value of 4.5 (Solvent A) and acetonitrile (Solvent B). The gradient systems used for the analysis are presented in Table 2. The detection of nadolol and the internal standard (metoprolol) were done at an excitation wavelength of 265 nm and an emission wavelength of 295 nm.

**Table 2:** HPLC-FL gradient profile for determination of nadolol in plasma.

Time (Min)	50 mM Ammonium acetate pH = 4.5 (%)	Acetonitrile (%)
00.0	93.0	07.0
0.50	93.0	07.0
6.00	80.0	20.0
9.00	80.0	20.0
14.00	40.0	60.0
15.00	40.0	60.0
15.10	93.0	07.0
18.00	93.0	07.0

The flow rate used was 1.2 mL/min. To develop a method for the quantification of nadolol in rat plasma, a standard solution was prepared and analyzed under varying parameters and chromatographic conditions, including injection volume, detection wavelength, flow rate, and gradient elution systems. Moreover, acetonitrile and perchloric acid were examined for their ability to provide the best peak shape, resolution, and detection response for direct protein precipitation extraction of plasma samples. The HPLC-FL method was validated in terms of selectivity, linearity, accuracy, precision, and recovery according to the US Food and Drug Administration (FDA) guidelines [34].

The method’s selectivity was assessed via analysis of plasma samples from six distinct blank rats to identify any potential interferences with the retention times of nadolol and metoprolol (IS). Plasma samples were spiked with nadolol at 1000 ng/mL. The linearity of the analytical method was evaluated using seven calibration standards, ranging in concentration from 2.5 to 1000 ng/mL. The six calibration curves were run on three separate days. Nadolol concentration was plotted against the peak areas’ ratios of nadolol/metoprolol. Linear regression analysis was performed to calculate nadolol concentrations. Data linearity was determined by examining the correlation coefficient (*r*^2^) and the y-intercept of the linear regression line. Deviation of ≤15% from the actual standard concentration was considered acceptable for the standard samples. Within- and between-run accuracy and precision were examined by preparing six replicates of nadolol in plasma at concentrations of 5 ng/mL (QCL), 100 ng/mL (QCM), 1000 ng/mL (QCH), and 2.5 ng/mL (LLOQ), that were analyzed over three days. The tested samples would pass if the precision calculated as the coefficient of variation (CV) of the QC samples is within ±15% and the LLOQ within ± 20% of the prepared concentration as per the US FDA guidelines. In addition, the accuracy was calculated as % deviation of the measured concentrations from the actual value and should be within a range of 85% to 115%. The recovery of nadolol was evaluated by spiking nadolol in plasma at three concentration levels: 5 ng/mL (QCL), 100 ng/mL (QCM), and 1000 ng/mL (QCH). Identical concentrations of nadolol in water were also prepared. Samples spiked in plasma were extracted and then analyzed. The peak area recoveries were compared with those spiked in water at the same concentration levels. The blank plasma samples used for method validation were obtained from untreated rats and were not part of the *in vivo* pharmacokinetic study.

### Pharmacokinetic parameters

Pharmacokinetic parameters were determined by analyzing nadolol plasma concentration-time data for each rat using the PK solver add-in for Microsoft Excel. The mean pharmacokinetic parameters were then calculated for each group [35]. A non-compartmental extravascular analysis was performed to estimate the following pharmacokinetic parameters: *C*_max_: maximum observed plasma concentration, *T*_max_: time to reach C_max_, *t*½: apparent terminal elimination half-life, AUC_0-t_: (partial AUC) area under the plasma concentration-time curve (AUC) from dosing (time 0) to time t, AUC_0-∞:_ the total AUC from dosing (time 0) extrapolated to infinite time, calculated using the following equation: ${AUC}_{\text{0}-\infty }={AUC}_{\text{0}-last}+\;\frac{C_{last}}{K_{e}}$ , CL/F: apparent oral clearance (CL/F) computed as Dose/AUC_0-∞_, V_z_/F: apparent volume of distribution during terminal phase (V_z_/F) after non-intravenous administration calculated as (CL/F)/*K*_e_, and MRT: mean residence time (MRT) is the average time that molecules of a dosed drug spend in the body.

### Phytochemical analysis of matcha

A high-performance liquid chromatography (HPLC) system with UV detection was used to measure caffeine in matcha green tea extract using an external calibration curve of an authentic reference standard. A stock solution of standard caffeine (100 mg/L) was prepared by accurately weighing and transferring 10 mg of caffeine to a 100 mL volumetric flask. Afterward, deionized water was gradually added, and the flask was shaken at room temperature to ensure maximum solubility. The caffeine stock solution was then diluted with deionized water to prepare a calibration curve consisting of six calibration concentrations (1, 2.5, 5, 7.5, 25, and 50 ppm) and three quality control points (12.5, 20, and 40 ppm).

About 100 g of matcha green tea powder was dissolved in 2 L of 80% ethanol. The mixture was left to shake in a water bath shaker at room temperature for 48 h. Afterward, about 10 *µ*L of the filtered supernatant was diluted with 990 *µ*L of deionized water and transferred into an HPLC vial for analysis. A Shimadzu prominence-i LC-2030C 3D Plus (Kyoto, Japan) connected with a PDA detector and a Rf HiQ sil C_18_-HS analytical column (4.6 × 250 mm, 5 *µ*m) (KYA Technologies, Tokyo, Japan) was used for chromatographic separation of caffeine. The mobile phase was a gradient blend consisting of 0.1% formic acid in deionized water (A) and acetonitrile (B) that increased linearly over 13 min from 100:0 (A: B) to 10:90 (A: B). The mobile phase was held at 90% B for 4 min, then returned to starting conditions (100% A) in 0.1 min, followed by a 3 min hold. Calibration standards and QC samples were injected in duplicate, while the matcha green tea sample was injected in quadruplicate in the 20-min run. The temperature of the HPLC column was set at 25 °C, the autosampler temperature was 8 °C, the flow rate used was 1 mL/min, the detection wavelength was 273 nm, and the injection volume was 20 *µ*L.

(-)-Epigallocatechin-3-gallate (EGCG), (–)-epicatechin, and (+)-catechin in the matcha green tea powder used in the present study were quantitatively determined as part of a separate, independently conducted study in our laboratory, using the same matcha batch [36]. Quantification was performed by a UPLC-HRESIMS method using authentic reference standards. Full analytical procedures are reported in the corresponding paper from that study [36].

### Statistical analysis

Data are presented as mean ± standard deviation (SD) except for *T*_max_, which is presented as the median with interquartile range (Q1-Q3). Plasma concentration-time profile figure is presented as mean ± standard error of mean (SEM). Nadolol pharmacokinetic parameters were analyzed using one-way ANOVA followed by Dunnett’s post hoc test for multiple comparisons. Statistical significance was set at *p* < .05. GraphPad Prism 8 (GraphPad Software Inc., San Diego, CA, USA) was used for carrying out statistical tests and for generating the graphs.

## Results

### Development and validation of HPLC-FL method for nadolol quantification

Details of the development and validation of the HPLC-FL method for nadolol quantification, including selectivity, linearity, accuracy, precision, and recovery, are provided in Supporting Information (Section 1). Method sensitivity was evaluated during validation; the limit of detection (LOD) for nadolol was 0.8 ng/mL, and the lower limit of quantification (LOQ) was 2.5 ng/mL, defined as the lowest concentration quantified with acceptable accuracy and precision in accordance with FDA bioanalytical method validation guidelines. Additional validation details are available in the Supporting Information.

### The effect of matcha on the pharmacokinetics of nadolol

**Fig 2:**
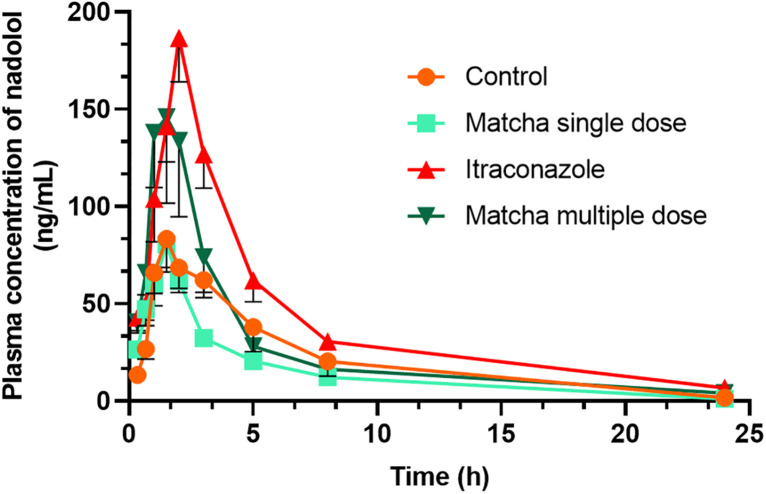
Plasma concentration-time profile of nadolol in rats following a single oral dose of 10 mg/kg nadolol administered at 30 min after Saline (Control, p.o. **), Matcha** **tea** **powder (250 mg/kg, p.o.), Itraconazole (50 mg/kg, p.o.), or with Matcha** **tea** **powder (250 mg/kg, p.o.) for 21** **days.** Plasma concentrations were measured at 0.33, 0.66, 1, 1.5, 2, 3, 5, 8, and 24 h. Data are presented as Mean ± SEM (n = 8).

Nadolol plasma concentration-time profiles for the four groups are depicted in Fig 2, and the corresponding pharmacokinetic parameters are summarized in Table 3. Log-transformed pharmacokinetic parameters yielded similar statistical results. Oral administration of 50 mg/kg of itraconazole significantly increased the AUC_0-t_ (IP group 974 ± 234 ng·h/mL versus NC group 513 ± 337 ng·h/mL, *p* = .009) and AUC_0-∞_ (IP group 1043 ± 200 ng·h/mL versus NC group 531 ± 336 ng·h/mL, *p* = .002) of nadolol in the positive control group (IP) compared with the control group (Fig 2 and Table 3). Although the *C*_max_ in the IP group increased by a factor of 1.9 and the Vz/F was reduced by ~33% in the IP compared with the NC group, their respective *p* values of.06 and.64 did not indicate significant differences between the two groups. On the other hand, itraconazole did not affect the median *T*_max_ (h) for nadolol. The *t*½ (h) increased non-significantly from 4.0 ± 1.6 h in the NC group to 6.4 ± 2.1 h in the IP (*p* = .279). A single oral dose of matcha (250 mg/kg) had no significant effect on the pharmacokinetic parameters of nadolol compared with the control group (*p* > .05, Fig 2 and Table 3). The matcha multiple-dose group exhibited a higher but not statistically significant *C*_max_ (183.8 ± 119.6 ng/mL for MM group vs. 101.3 ± 42.3 ng/mL for the NC group, *p* = .127), AUC_0-t_ (623 ± 315 ng·h/mL for MM group vs. 513 ± 337 ng·h/mL, *p* = .841), and AUC_0-∞_ (683 ± 305 ng·h/mL for the MM group vs. 531 ± 336 ng·h/mL for the NC group, *p* = .636) for nadolol as compared with the NC group (Fig 2 and Table 3). In contrast, *t*½ (h) significan*t*ly increased to 7.7 ± 4.2 from 4.0 ± 1.6 in the control group, *p* = .039. In summary, chronic administration of matcha for 21 days (250 mg/kg/day) had no significant effect on the pharmacokinetic profile of nadolol, except for a significant increase in the *t*½.

**Table 3:** Pharmacokinetic parameters of nadolol in rat plasma following a single oral dose of 10 mg/kg nadolol, which was administered at 30 min after normal saline (Control, p.o.), Matcha tea powder (250 mg/kg, p.o.), Itraconazole (50 mg/kg, p.o.), or with Matcha tea powder (250 mg/kg, p.o.) for 21 days (*n* = 8).

PK parameters^a^	Control^b^	Matcha single dose^b^	Itraconazole^b^	Matcha multiple dose^b^
*C*_max_ (ng/mL)	101.3 ± 42.3	85.4 ± 37.0	194.5 ± 58.6	183.8 ± 119.6
*T*_max_ (h)	2.0 (1.5–3.0)^c^	1.5 (1.4–1.6)^c^	2.0 (2.0–2.0)^c^	1.5 (1.4–2.0)^c^
*t*½ (h)	4.0 ± 1.6	4.4 ± 2.0	6.4 ± 2.1	7.7 ± 4.2*^d^
AUC_0-t_ (ng·h/mL)	513 ± 337	332 ± 122	974 ± 234**^d^	623 ± 315
AUC_0-∞_ (ng·h/mL)	531 ± 336	364 ± 122	1043 ± 200**^d^	683 ± 305
MRT (h)	5.4 ± 1.5	5.7 ± 1.8	7.8 ± 3.1	8.0 ± 3.8
Vz/F (mL/kg)	143 ± 82	176 ± 71	96 ± 48	180 ± 110
CL/F (mL/h.kg)	29.0 ± 2.4	30.7 ± 11.4	9.9 ± 2.1	16.3 ± 4.6

^a^ *C*_max_: maximum plasma concentration; *T*_max_: time to reach *C*_max_; *t*½: apparent terminal elimination half-life; AUC_0-t_: (partial AUC) area under the concentration-time curve (AUC) from dosing (time 0) to time t; AUC_0-∞:_ the total AUC from dosing (time 0) extrapolated to infinite time; MRT: mean residence time; V_z_/F: apparent volume of distribution during terminal phase after non-intravenous administration; CL/F: apparent oral clearance

^b^ Data are presented as mean ± standard deviation (SD)

^c^ Data for *T*_max_ is presented as median and interquartile ranges (25%−75%) (n = 8)

^d^ * *p* < .05 and ** *p* < .01 compared with control.

### Phytochemical analysis of matcha

Caffeine, a key marker compound in matcha, was quantified using an HPLC-UV and found to be 4.18 ± 0.44% w/w of dry matcha powder (41.8 ± 4.4 mg/g). Additional details on the HPLC-UV method used for caffeine quantification are provided in Supporting Information, Section 2.

(–)-Epigallocatechin-3-gallate (EGCG), (–)-epicatechin, and (+)-catechin were quantitatively determined using UPLC-HRESIMS. As summarized in Table 4, EGCG was the predominant catechin, present at 1997 ± 49 *µ*g/g dry weight, whereas (–)-epicatechin and (+)-catechin were detected at 4.05 ± 0.34 *µ*g/g and 0.23 ± 0.01 *µ*g/g, respectively.

**Table 4:** Quantitative catechin profile of the matcha green tea powder.

Compound	Content (*µ*g/g dry weight)^a^
(−)-Epigallocatechin-3-gallate	1997 ± 49
(+)-Catechin	0.23 ± 0.01
(−)-Epicatechin	4.05 ± 0.34

^a^Values are expressed as mean ± SD (n = 3).

## Discussion

This study is the first to investigate the effect of single and multiple doses of matcha on the pharmacokinetics of nadolol in male Sprague-Dawley rats. A validated HPLC-FL method was used to quantify nadolol in plasma samples. Additionally, an HPLC-UV method was used for quantitative analysis of caffeine in matcha extract.

In the control group, rats received an oral dose of nadolol (10 mg/kg) 30 min after pretreatment with normal saline. The *C*_max_, AUC_0-t_, and AUC_0-∞_ were found as 101.3 ± 42.3 ng/mL, 513 ± 337 ng·h/mL, and 531 ± 336 ng·h/mL, respectively. The *t*½ and median *T*_max_ values were 4.0 and 2.0 h, respectively. The results obtained were in good agreement with those reported in the literature. For example, Miyazaki et al (2013) reported values of 122 ± 14 ng/mL, 212 ± 17 ng·h/mL, 297 ± 28 ng·h/mL, 1.5 h, and 2.0 h for *C*_max_, AUC_0–3_, AUC_0-∞_, median *T*_max_, and *t*½, respectively, after oral administration of 10 mg/kg of nadolol [30]. In another study by Misaka et al (2013), using the same oral dose, the *C*_max_, AUC_0-∞_, median *T*_max_, and *t*½ values were reported as 168 ± 28 ng/mL, 433 ± 69 ng·h/mL, 2.0 h, and 2.1 h, respectively [26].

Itraconazole, an anti-fungal agent, is a potent inhibitor of P-gp and CYP3A [37]. In this study, itraconazole was used as a positive control to confirm the validity of the experimental animal model for P-gp inhibition. Pretreatment with itraconazole (50 mg/kg) 30 min prior to nadolol administration significantly increased nadolol AUC_0-∞ (_~96%) and AUC_0-t_ (~90%) compared with the control group. Median *T*_max_ remained unchanged following itraconazole administration, while the *C*_max_ and *t*½ were non-significantly increased. Itraconazole is a potent inhibitor of P-gp and CYP3A [37]. On the other hand, nadolol is not metabolized by CYP450 enzymes but it is a substrate for P-gps [29,30]. The observed increase in nadolol AUC following itraconazole administration can be attributed to P-gp inhibition in the apical membrane of the intestinal epithelium, reducing nadolol efflux and increasing its systemic exposure. These findings align with Miyazaki et al. (2013), who reported that P-gp inhibition by itraconazole enhances the bioavailability of oral nadolol [30]. A prior clinical trial showed that itraconazole substantially raised the AUC value of celiprolol, presumably because itraconazole inhibits intestinal P-gp [38]. Another study showed that itraconazole inhibitory effects for the typical P-gp substrates verapamil and prazosin were comparable in human and rat P-gp, indicating that itraconazole inhibition of P-gp is species-independent [39]. Our findings agreed with the literature indicating that oral nadolol exhibits a higher bioavailability since itraconazole inhibits the P-gp in the intestine.

A single dose of matcha (250 mg/kg) resulted in a slight but statistically insignificant reduction in nadolol *C*_max_, AUC_0-t_, and AUC_0-∞_. Additionally, other pharmacokinetic parameters did not differ significantly between the matcha and control groups. These findings suggest that a single dose of matcha has no significant effect on nadolol pharmacokinetics. A single dose of matcha green tea may not reach the threshold concentration necessary to significantly inhibit P-gp activity. Furthermore, the slight reduction in nadolol exposure may be due to a small increase in volume of distribution, as total clearance remained unchanged between the two groups.

Chronic administration of matcha (250 mg/kg for 21 days) increased nadolol *C*_max_ by ~81% and AUC_0–∞_ by ~29%, though these changes were not statistically significant. However, *t*½ was significantly prolonged. This pattern can occur after oral dosing because AUC is governed primarily by F/CL, whereas *t*½ depends on the terminal slope (λz) and the ratio Vz/CL (*t*½ = 0.693 × Vz/CL) [40]. In the multiple-dose matcha group, CL/F was numerically lower and Vz/F numerically higher than in the control group, a combination expected to prolong *t*½. Moreover, terminal-phase parameters are more sensitive to interindividual variability and λz estimation than AUC, which can result in statistically significant changes in *t*½ despite modest, non-significant changes in AUC [40]. Previous studies have shown that intestinal P-gp inhibition increases the plasma concentration of P-gp substrates such as talinolol [41]. Similarly, itraconazole-mediated suppression of intestinal P-gp significantly increased nadolol plasma concentrations [30]. The current findings are consistent with the outcome of a pharmacokinetic interaction caused by P-gp inhibition. However, they contradict a study by Misaka et al. (2013), which reported that pretreatment with green tea extract significantly reduced nadolol AUC (by ~74%) and *C*_max_ (by ~85%) [26]. When compared to the control group, terminal elimination half-life (*t*½) of nadolol was significantly increased in multiple-dose matcha group, which may be partially attributed to a reduction in total clearance. The overall decrease in nadolol elimination in the multiple-dose matcha group may be due to P-gp inhibition, which reduced biliary excretion of nadolol [42]. A previous study reported that epigallocatechin gallate (EGCG) pretreatment reduced biliary elimination of the cancer drug irinotecan (CPT-11) and its active metabolite SN-38, leading to a significant increase in plasma half-life and AUC [43]. The apical membrane of the renal proximal tubule cells as well as hepatocytes both express P-gp [44]. In addition to P-gp inhibition, immunoblots of HK-2 cells grown in the presence of EGCG showed a significant reduction in P-gp expression [45]. The present study demonstrated that the administration of multiple doses of matcha for 21 days increased the plasma concentration of nadolol in rats. However, the increases were not statistically significant. The overall observed effect could be partially explained by the inhibition of P-gp in the intestine as well as in the biliary duct.

The differences between our findings and those of Misaka et al. (2013) are likely attributable to differences in green tea formulation and dosing regimen [26]. Misaka et al. administered green tea extract (400 mg/kg) or purified EGCG (150 mg/kg) as a single pretreatment, resulting in high catechin exposure and marked reductions in nadolol *C*_max_ and AUC consistent with inhibited intestinal uptake [26]. In contrast, the present study used whole-leaf matcha powder (250 mg/kg), providing EGCG within a complex phytochemical matrix and under both single and repeated dosing conditions, and did not observe a pronounced reduction in nadolol systemic exposure. These findings indicate that green tea–nadolol interactions are formulation- and context-dependent, and that effects observed with extracts or isolated catechins may not directly translate to nutritionally relevant whole-leaf matcha consumption.

In the present study, quantitative phytochemical analysis confirmed that the matcha batch used contained measurable levels of epigallocatechin-3-gallate (EGCG), the catechin most frequently implicated in green tea–nadolol interactions [26,32,46,47]. Despite the presence of EGCG, repeated administration of whole-leaf matcha did not result in a marked reduction in nadolol systemic exposure. This observation contrasts with reports using green tea extracts or isolated EGCG and underscores that the pharmacokinetic impact of green tea products on nadolol is formulation- and context-dependent. The complex phytochemical matrix of whole-leaf matcha and the effects of repeated intake may modulate transporter-mediated interactions differently from acute exposure to purified catechins, highlighting the importance of evaluating herb–drug interactions using preparations and dosing patterns that reflect real-world consumption.

Functional pharmacodynamic parameters such as heart rate were not assessed in the present study, which was designed primarily to evaluate pharmacokinetic interactions. The modest and largely non-significant changes in nadolol systemic exposure following matcha administration suggest that clinically meaningful functional effects are unlikely under the conditions studied. Future investigations integrating pharmacokinetic and pharmacodynamic endpoints may further clarify the clinical relevance of matcha–nadolol coadministration.

## Conclusions

This study is the first to investigate the potential pharmacokinetic interaction between matcha and nadolol, providing new insights into the effects of matcha consumption on drug absorption and disposition. The findings suggest that single and multiple oral doses of matcha do not significantly alter the pharmacokinetics of nadolol, indicating minimal effects on nadolol’s absorption, distribution, or elimination. However, given the complexity of drug-nutrient interactions and the potential variability in individual responses, further research is needed to confirm these findings. Future studies should investigate how different formulations, dosages, and durations of matcha consumption affect nadolol pharmacokinetics. Additionally, research should assess interindividual variability, particularly the role of genetic polymorphism and underlying health conditions in modulating nadolol metabolism. A more comprehensive understanding of these interactions is crucial before establishing definitive clinical recommendations regarding matcha and nadolol coadministration, particularly in cardiovascular patients who rely on stable drug levels for effective treatment.

## Supporting information

S1 File Methods and analytical data. (DOCX)

S2 Data Nadolol pharmacokinetics. (XLSX)
