# Supplementary material for: Effects of matcha green tea on the pharmacokinetics of nadolol in rats
Source: PLoS One. 2026 Feb 13;21(2):e0342857. doi: 10.1371/journal.pone.0342857 (PMC12904406; doi:10.1371/journal.pone.0342857)
Supplement: S1 File — (DOCX) [file pone.0342857.s001.docx]

**Supporting Information**

**Effects of matcha green tea on the pharmacokinetics of nadolol in rats**

Eslam T. Mashaqbeh^1^, Tamam El-Elimat^1*^, Osama Y. Alshogran^2^, Iyad Hamzeh^2^, Zahraa M. Obeidat^1^, Ahmed H. Al Sharie^3^, Feras El Hajji^4^

^1^ Department of Medicinal Chemistry and Pharmacognosy, Faculty of Pharmacy, Jordan University of Science and Technology, Irbid, Jordan

^2^ Department of Clinical Pharmacy, Faculty of Pharmacy, Jordan University of Science and Technology, Irbid, Jordan

^3^ Department of Internal Medicine, Southeast Health, Dothan, Alabama, United States of America

^4^ Department of Clinical Pharmacy and Therapeutics, Faculty of Pharmacy, Applied Science Private University, Amman, Jordan

^*^ Corresponding author

E-mail: telimat@just.edu.jo (TE)

**Section 1: Development and Validation of HPLC-FL Method for Nadolol Quantification:**

**Section 1A: Selectivity:**

An HPLC-FL method was developed to quantify nadolol in plasma and urine samples. For plasma samples, nadolol and metoprolol (IS) were eluted at 8.6 and 12.9 min, respectively (**S1B Fig**). The developed method was validated in terms of selectivity, linearity, recovery, precision, and accuracy as per the US Food and Drug Administration guidelines. The selectivity of the method was confirmed by the absence of any detectable interferences from plasma and urine within the defined retention times of both nadolol and metoprolol (IS) using six blank rat plasma (**S1B Fig**).

| A) |
| --- |
| 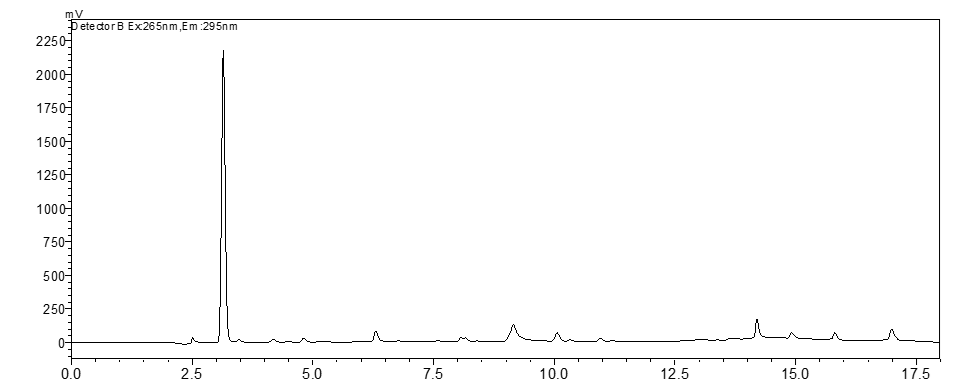 |
| B) |
| 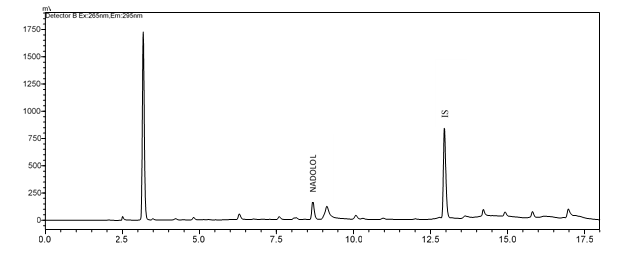 |

**S1 Fig. HPLC Chromatograms of (A) Blank Plasma; (B) Plasma Spiked with Nadolol at a Concentration of 1000 ng/mL (*t*_R_ = 8.6 min) and IS (*t*_R_ = 12.9 min).**

**Section 1B: Linearity:**

To test the linearity of the calibration curves of nadolol under the optimized conditions, six calibration curves at seven concentration levels 2.5, 5, 10, 50, 100, 500, and 1000 ng/mL of nadolol in plasma were prepared and injected over three days. Nadolol-to-IS peak areas ratios were plotted against the respective authentic nadolol concentration. Excellent linearity was obtained as evidenced by a correlation coefficient (*r*^2^) of 0.9990-0.9999 for all calibration curves (**S1** **Table and S2 Fig**). The limit of detection (LOD) was 0.8 The limit of quantification (LOQ) was 2.5 and 50 ng/mL.

| **S1 Table. Calibration Curve Parameters for Nadolol in Rat Plasma (*n* = 6).** | | | | |
| --- | --- | --- | --- | --- |
| **Calibration curve #** | **Range (ng/L)** | **Slope** | **Intercept** | **Correlation coefficient** |
| 1 | 2.5-1000 | 0.0002 | 0.00448 | 0.9990 |
| 2 |  | 0.0005 | 0.00125 | 0.9993 |
| 3 |  | 0.0002 | 0.00096 | 0.9998 |
| 4 |  | 0.0005 | 0.0029 | 0.9994 |
| 5 |  | 0.0004 | 0.0030 | 0.9995 |
| 6 |  | 0.0005 | 0.0025 | 0.9999 |
| Mean |  | 0.00038 | 0.0252 | 0.9995 |
| SD |  | 0.0001 | 0.0013 | 0.00033 |
| SD: Standard deviation. | | | | |

| 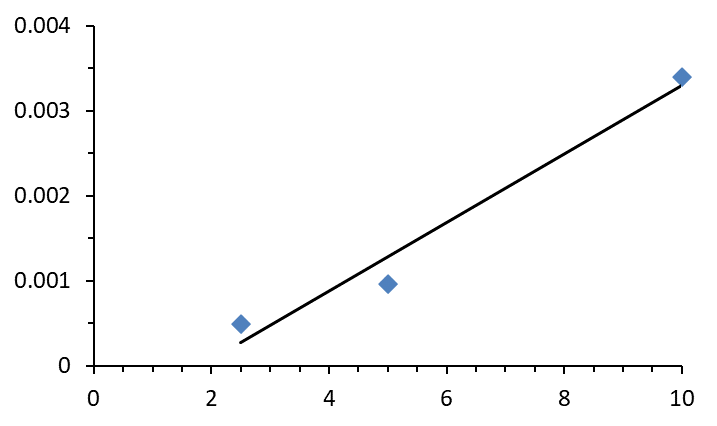 |
| --- |

**S2 Fig. Representative Calibration Curves for Nadolol Measured by HPLC-FL at 2.5, 5, 10, 50, 100, 500, and 1000 ng/mL Concentration Levels in Plasma**.

**Section 1C: Precision, accuracy, and recovery:**

Accuracy and precision were measured using the four QC points (QCH, QCM, QCL, and LLOQ) analyzed in 6 replicates for three consecutive days. Precision and accuracy were evaluated by calculating within-run (**S2 Table**) and between-run (**S3 Table**) variations of QC samples in six replicates. Accuracy was checked as percent recovery for each QC sample. The precision was checked as percent of coefficient variation (CV %). The within-run precision (CV %) and accuracy (% recovery) ranged from 2.79 to 11.9 and 100.50 to 103.92%, respectively (**S2 Table**). Similarly, between-run precision and accuracy ranged from 1.49 to 8.70 and 95.60 to 100.59%, respectively (**S3 Table**). In accordance with US Food and Drug Administration guidelines acceptance criteria, the methods were found to be accurate and precise within the analyzed ranges. To evaluate the methods’ recovery, a known quantity of nadolol was spiked into plasma. The recovery of nadolol from plasma was 97.2, 96.7, and 100.3% at spiked concentrations of 5, 100, and 1000 ng/mL, respectively; confirming the efficient recovery of the drug.

| **S2 Table. Within-Run Precision and Accuracy of The Method for Determination of Nadolol in Plasma (*n* = 6).** | | | | | |
| --- | --- | --- | --- | --- | --- |
| **QC level** | **Nominal conc. (ng/mL)** | **Measured Mean conc. (ng/mL)** | **SD** | **CV%** | **% Recovery** |
| QCH | 1000 | 1013.34 | 28.25 | 2.79 | 101.33 |
| QCM | 100 | 100.5 | 3.51 | 3.49 | 100.50 |
| QCL | 5 | 5.12 | 0.36 | 7.03 | 102.40 |
| LLOQ | 2.5 | 2.598 | 0.31 | 11.9 | 103.92 |
| QC: Quality Control, QCH: Quality Control High, QCM: Quality Control Medium, QCL: Quality Control Low, LLOQ: Lower Limit of Quantification, Nominal conc.: Nominal Concentration, Measured Mean conc.: Measured Mean Concentration, SD: Standard Deviation, CV%: Coefficient of Variation (%), % Recovery: Percent Recovery. | | | | | |

| **S3 Table. Between-Run Precision and Accuracy of The Method for Determination of Nadolol in Plasma (*n* = 18).** | | | | | |
| --- | --- | --- | --- | --- | --- |
| **QC level** | **Nominal conc. (ng/mL)** | **Measured Mean conc. (ng/mL)** | **SD** | **CV%** | **% Recovery** |
| QCH | 1000 | 1005.9 | 14.96 | 1.49 | 100.59 |
| QCM | 100 | 98.93 | 6.3 | 6.37 | 98.93 |
| QCL | 5 | 4.83 | 0.42 | 8.70 | 96.6 |
| LLOQ | 2.5 | 2.39 | 0.19 | 7.95 | 95.60 |
| QC: Quality Control, QCH: Quality Control High, QCM: Quality Control Medium, QCL: Quality Control Low, LLOQ: Lower Limit of Quantification, Nominal conc.: Nominal Concentration, Measured Mean conc.: Measured Mean Concentration, SD: Standard Deviation, CV%: Coefficient of Variation (%), % Recovery: Percent Recovery. | | | | | |

**Section 2: Phytochemical analyses of Match tea:**

A calibration curve of an authentic reference standard of caffeine was prepared to calculate caffeine content in matcha tea using HPLC. Caffeine eluted at 6.9 min (**S3 Fig**). Linear calibration curve was constructed with *r*^2^ value of 0.9986 in the range of 1-50 *µ*g/mL (1, 2.5, 5, 7.5, 25, and 50 ppm) as shown in **S4 Fig**. Three quality control points at 12.5, 20, and 40 ppm were accurate within -3.7%, 6.94%, and -3.17% (ppm, RSD %) from actual concentration as shown in **S4 Table**. Matcha tea sample was injected in quadruplicate, the results were analyzed, and the percent of caffeine content was calculated against external caffeine standard using the following equation:

% w/w caffeine = C × FV × D × 100% / W

Where C is the sample’s caffeine concentration (mg/mL), extrapolated from the calibration curves’ linear regression, FV is the final volume of the sample in mL, D is the dilution factor, and W is the sample weight in mg. The caffeine content of matcha was calculated as 4.18 ± 0.44% mg/mg of dry powder of matcha green tea. **Figure S5** shows the HPLC chromatogram of the matcha tea extract that was used to measure the caffeine content.

**S3 Fig. HPLC Chromatogram of Caffeine Standard (7.5 ppm); Caffeine Eluted at 6.9 min.**

| **S4 Table. Quality Control Points for The Calibration Curve**. | | |
| --- | --- | --- |
| **Actual Concentration^a^** | **Measured Concentration (*µ*g/mL)** | **%Error** |
| 12.5 | 12.03 | -3.76 |
| 20 | 21.39 | 6.95 |
| 40 | 38.73 | -3.17 |
| ^a^ Within run precision *n* = 2. %Error: percent error | | |


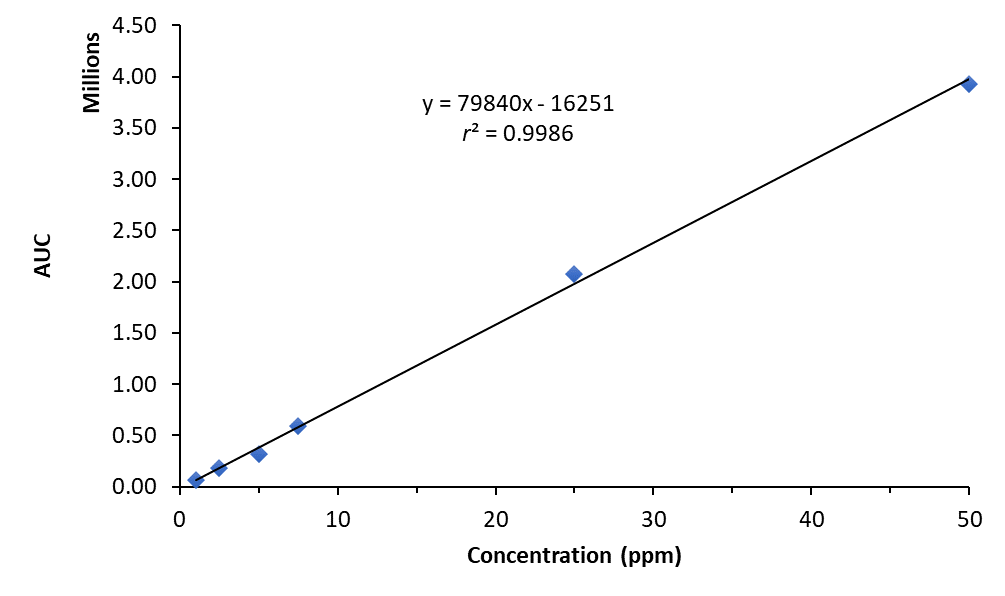


**S4 Fig. Standard Calibration Curve of Caffeine Obtained at Six Concentration Levels (1, 2.5, 5, 7.5, 25, and 50 ppm).**

**S5 Fig. HPLC Chromatogram of Matcha Tea Extract.**
